# Anxiety, Depression, and Stress Reaction/Adjustment Disorders and Their Associations with Healthcare Resource Utilization and Costs Among Newly Diagnosed Patients With Breast Cancer

**DOI:** 10.36469/001c.70238

**Published:** 2023-03-28

**Authors:** Dingwei Dai, Henriette Coetzer, Sean R. Zion, Michael J. Malecki

**Affiliations:** 1 CVS Health Clinical Trial Services, LLC, Woonsocket, Rhode Island, USA; 2 Blue Note Therapeutics, Inc., San Francisco, California, USA

**Keywords:** cancer-related distress, anxiety, depression, adjustment disorder, healthcare resource utilization, healthcare costs, claims database analysis

## Abstract

**Background:** Breast cancer is the most common cancer among women in the United States. Newly diagnosed patients with breast cancer often experience anxiety, depression, and stress. However, the impact of psychological distress on healthcare resource utilization (HCRU) and costs has not been adequately assessed.

**Objectives:** To evaluate the incidence and prevalence of anxiety, depression, and stress reaction/adjustment disorder among patients newly diagnosed with breast cancer, to examine HCRU and costs, and to assess the association of these psychiatric disorders with costs.

**Methods:** This retrospective observational cohort study was conducted using a large US administrative claims database with an index date of newly diagnosed breast cancer. Demographics and comorbidities (including anxiety, depression, and stress reaction/adjustment disorder) were assessed using data collected 12 months before and after the index date. HCRU and costs were assessed using data collected 12 months after the index date. Generalized linear regressions were performed to examine the association between healthcare costs and anxiety, depression, and stress reaction/adjustment disorder.

**Results:** Of 6392 patients with newly diagnosed breast cancer, 38.2% were diagnosed with psychiatric disorders including anxiety (27.7%), depression (21.9%), or stress reaction/adjustment disorder (6%). The incidence of these psychiatric disorders was 15% and the prevalence was 23.2%. Patients with anxiety, depression, or stress reaction/adjustment disorder had higher rates of several types of HCRU (*P* < .0001) and higher total all-cause costs compared with patients without these psychiatric disorders (*P* < .0001). Patients with incident anxiety, depression, or stress reaction/adjustment disorder incurred higher all-cause costs in the first year following breast cancer diagnosis than those with prevalent anxiety, depression, or stress reaction/adjustment disorder (*P* < .0003), or those without these psychiatric disorders (*P* < .0001).

**Discussion:** Of patients with anxiety, depression, or stress reaction/adjustment disorder, those with incident psychiatric disorders had higher healthcare costs, suggesting that new-onset psychological distress may contribute to higher costs incurred by the payer. Timely treatment of psychiatric disorders in this population may improve clinical outcomes and reduce HCRU and costs.

**Conclusions:** Anxiety, depression, and stress reaction/adjustment disorder were common among patients newly diagnosed with breast cancer and were associated with increased healthcare costs in the first year following breast cancer diagnosis.

## BACKGROUND

Breast cancer is the most common cancer among women in the United States, accounting for about 30% of all new female cancers recorded each year.^1^ With advancements in early screening, diagnosis, and breakthrough treatments, 5-year breast cancer survival rates continue to improve, ranging from 40% in low-income countries to 80% in developed countries.^2^ Although the quality of life of patients with breast cancer has also improved over time,^3,4^ quality of life is lower for patients undergoing treatment compared with those who have completed treatment,^3^ likely due to various treatment-related symptoms (eg, pain, worry, sexual dysfunction).^4^

Patients with cancer also commonly experience anxiety, depression, and/or stress reaction/adjustment disorder; however, the reported prevalence of these forms of distress varies widely.^5^ In patients with early-stage breast cancer, the prevalence of moderate to severe anxiety, depression, and stress was found to be 26.2%, 31.8%, and 36%, respectively.^6^ The emotional distress and associated psychiatric disorders experienced by patients with cancer often go unrecognized and untreated by healthcare providers,^7^ likely due to limited time and insufficient training in screening and management of emotional distress and related disorders.^8,9^ Nevertheless, distress and psychiatric disorders affect a range of key oncology health outcomes. These include lower medication adherence and persistence, and treatment engagement and effectiveness, which can increase both morbidity and mortality.^10,11^

Clinical guidelines for the treatment of anxiety, depression, and adjustment disorders in patients with cancer recommend a comprehensive approach to care that incorporates both pharmacologic and psychosocial interventions.^12–14^ Pharmacologic decision-making should balance benefits of treatment with the potential for adverse effects, poor tolerability, and possible drug interactions.^12^ Antidepressant medication may be reserved for patients with more moderate to severe symptoms, with a focus on selective serotonin reuptake inhibitors due to their tolerability and lower potential for drug interactions.^13^ However, antidepressants that are potent CYP2D6 inhibitors are not recommended in combination with tamoxifen,^13^ and long-term use of benzodiazepines for the treatment of anxiety should also be avoided.^12^ Psychosocial interventions that are recommended by oncology clinical guidelines range from low-intensity interventions, such as psychoeducation, self-help programs, and group-based peer support, to more structured evidence-based treatments, such as behavioral activation, cognitive behavioral therapy, and interpersonal therapy.^12–14^

Psychological distress creates an increased economic burden on patients and the healthcare system,^15,16^ which currently lacks the resources to address the psychological needs of patients with cancer. Specifically, adequate psychological staffing in cancer centers is highly variable, with an average of 2523 newly registered patients per year for each full-time psychologist.^17^ Moreover, patients report poor access to specialized psycho-oncological care.^18^ Due to limited access to appropriate care, the economic burden is a consequence of increased healthcare resource utilization (HCRU) in other areas, such as emergency department (ED) visits, outpatient visits, inpatient admissions, mental health services, and primary care physician/provider (PCP) visits.^15,16^

This article reports on the incidence and prevalence of anxiety, depression, and stress reaction/adjustment disorders among newly diagnosed patients with breast cancer and investigates their impact on HCRU and healthcare costs.

### Objectives

Currently, there is limited real-world evidence regarding the impact of anxiety, depression, and stress reaction/ adjustment disorder among newly diagnosed patients with breast cancer on HCRU and healthcare costs. Therefore, the objectives of this study were: (1) to evaluate the incidence and prevalence of anxiety, depression, and stress reaction/ adjustment disorder among patients newly diagnosed with breast cancer; (2) to examine all-cause HCRU and costs, and mental health–related HCRU and costs, among patients newly diagnosed with breast cancer, and compare them between patients with vs without psychiatric disorders and between patients with incident vs prevalent psychiatric disorders; and (3) to assess the association between healthcare costs and anxiety, depression, and stress reaction/adjustment disorder among patients newly diagnosed with breast cancer.

## METHODS

### Study Design

This was a retrospective observational cohort study of newly diagnosed patients with breast cancer using Aetna administrative claims data from January 1, 2017, to December 31, 2020 (study period), representing one part of a retrospective study of comorbid psychiatric disorders in breast cancer within a managed care system. Details of the study design and data source have been published previously.^19^ Detailed inclusion and exclusion criteria are described in **Supplementary Figure S1**. The database includes over 20 million Aetna members and contains patient-level data including inpatient and outpatient data, and medical and pharmacy claims of fully insured commercial health plan and Medicare Advantage members. All data handling complied with federal and state requirements, and the privacy and security of individually identifiable personal health information were preserved as required by Health Insurance Portability and Accountability Act (HIPAA) standards. The study was approved by the Sterling Institutional Review Board (Atlanta, Georgia) prior to initiation. The reporting of this study conforms to the STROBE guidelines for observational cohort studies.^20^

**Figure 1.**
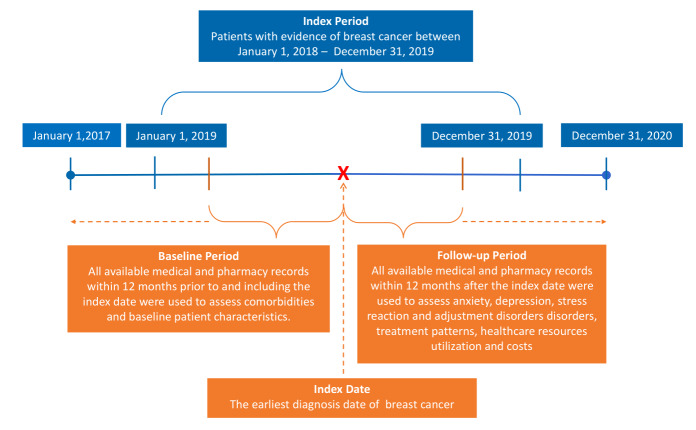
Study Design and Time Frame

Patients with breast cancer were identified between January 1, 2018, and December 31, 2019 (index period), by *International Classification of Diseases, Tenth Revision, Clinical Modification* (ICD-10-CM: C50.x) codes. The index date was defined as the earliest diagnosis date of breast cancer within the index period. The baseline period consisted of at least 12 months of continuous medical and pharmacy coverage prior to the index date, and the follow-up period consisted of at least 12 months of continuous medical and pharmacy coverage post-index date (**Figure 1**). Breast cancer was defined as (1) 1 hospitalization with case-defining breast cancer (ICD-10-CM: C50.x) in the first diagnostic position (primary diagnosis), or (2) 1 hospitalization with a Z code indicating radiotherapy (Z51.12) in the first diagnostic position and any case-defining diagnosis of breast cancer (C50.x) in the second diagnostic position, or (3) 3 or more outpatient medical encounters occurring within a 90-day period with any case-defining diagnoses of breast cancer (C50.x) in the first or second diagnostic position.^19^

The demographic characteristics of patients included age at index date; US geographic region (Midwest, Northeast, South, and West); rural, suburban, or urban residence; and median household income. Household income was estimated by merging 2010 US Census data with the claims data using zip codes. Comorbid conditions were assessed using all available data (any inpatient and outpatient claims) prior to and including the index date. All conditions were defined using previously described methods based on ICD-10-CM codes.^21–23^ The Charlson Comorbidity Index,^24^ a validated approach that summarizes disease burden and predicts risks of mortality and high healthcare costs,^25,26^ was also calculated.

Anxiety (F40.x [phobic anxiety disorders], F41.x [other anxiety disorders]), depression (F32.x [major depressive disorder, single episode], F33.x [major depressive disorder, recurrent]), and stress reaction/adjustment disorder (F43.x [reaction to severe stress, and adjustment disorders]) were identified using ICD-10-CM codes in the claims data. Incident cases were defined as patients with anxiety, depression, or stress reaction/adjustment disorder during the 1-year follow-up period, without any diagnosis of anxiety, depression, or stress reaction/adjustment disorder during the 1-year baseline period. Prevalent cases were defined as patients with any evidence of anxiety, depression, or stress reaction/adjustment disorder in the 1-year follow-up period and the 1-year baseline period.

All-cause HCRU and costs were assessed using all medical claims and pharmacy claims and were aggregated over the 12-month follow-up period. HCRU included any ED visits, outpatient visits, mental health services, inpatient admissions, and length of stay (LOS) among those with inpatient admissions. Mental health–related HCRU and costs were assessed using medical claims identified with a primary diagnosis of anxiety, depression, or stress reaction/adjustment disorder, or pharmacy claims identified with depression-, anxiety-, or stress reaction/adjustment disorder–related medications within the follow-up period. All healthcare costs reported in this study refer to allowed costs (total amount paid by the payer), and were inflated to 2020 US dollars using the medical care services component of the Consumer Price Index.^27^

### Statistical Analysis

Continuous and categorical data were expressed as means (± SD) and relative frequencies (%), respectively. Statistical significance was assessed with Student’s *t*-test, or Wilcoxon rank-sum test for continuous variables, and χ^2^ tests for categorical variables. To evaluate the association between anxiety, depression, and stress reaction/adjustment disorder and healthcare costs, multivariable generalized linear regression models were performed to calculate adjusted cost ratios with corresponding 95% confidence intervals and incremental cost differences. Age, geographic region, rural/suburban/urban residence, household income, payer type, surgery year, number of comorbid conditions, number of cancer-related therapies, and previous year total all-cause healthcare costs were controlled for. Log-transformation and gamma distribution were applied based on the distribution and presence of heteroskedasticity. Log link was chosen as it has been found to best specify the relationship between cost and explanatory variables.^28^ All *P* values were 2-sided, with *P* < .05 considered statistically significant. All data management and statistical analyses were conducted using SAS version 9.4 statistical software (SAS Institute Inc., Cary, North Carolina).

## RESULTS

A total of 6392 patients with newly diagnosed breast cancer were included in the study (**Supplementary Figure S1**). The average (SD) age was 67.43 (12.23) years. The baseline median household income, percentage of commercial and Medicare insurance payers, and the number of comorbid conditions are presented in the baseline patient characteristics (**Supplementary Table S1**).

### Objective 1: Incidence and Prevalence of Comorbid Psychiatric Disorders

**Figure 2.**
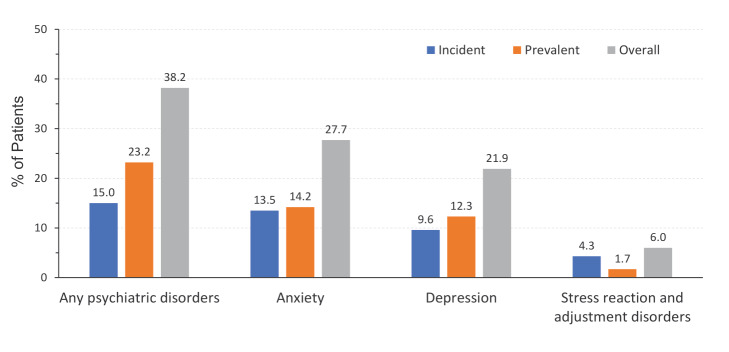
Incidence and Prevalence of Psychiatric Disorders Among Newly Diagnosed Patients With Breast Cancer Any psychiatric disorders include anxiety, depression, and stress reaction/adjustment disorders.

In this population, the incidence of anxiety, depression, or stress reaction/adjustment disorder was 15% (n = 959), and the prevalence was 23.2% (n = 1485). Overall (including both incident and prevalent cases), 38.2% (n = 2444) of patients with newly diagnosed breast cancer experienced anxiety, depression, or stress reaction/adjustment disorder during the 1-year follow-up period. Anxiety was the most common of the 3 types of psychiatric disorders, observed in 27.7% of patients during the first year following breast cancer diagnosis. Depression was observed in 21.9% of patients, and stress reaction/adjustment disorder was observed in just 6% of patients (**Figure 2**).

### Objective 2: All-Cause and Mental Health–Related HCRU and Costs

During the first year following breast cancer diagnosis, patients with anxiety, depression, or stress reaction/adjustment disorder were significantly more likely to see their PCP (94.84% vs 88.98%, *P* < .0001), visit the ED (34.12% vs 22.92%; *P* < .0001), and incur an inpatient admission (28.31% vs 18.29%; *P* < .0001) compared with patients without these psychiatric disorders. They also had significantly longer LOS for inpatient admissions (mean [SD] days: 2.23 [6.28] vs 1.06 [3.99]; *P* < .0001) compared with patients without anxiety, depression, or stress reaction/adjustment disorder. Additionally, patients with anxiety, depression, or stress reaction/adjustment disorder were more likely to see a specialist (99.14% vs 97.44%, *P* < .0001), have a psychiatry visit (5.32% vs 0.20%, *P* < .0001), and engage in any mental health services (16.65% vs 0.73%, *P* < .0001) than patients without these psychiatric disorders (**Table 1**).

**Table 1.** Healthcare Resource Utilization Among Patients With Breast Cancer in the First Year Following Diagnosis

	**No Psychiatric Disorders (n = 3948)**	**Psychiatric Disorders (n = 2444)**	***P* Value^a^**	**Psychiatric Disorders**		
				**Incident (n = 959)**	**Prevalent (n = 1485)**	***P* Value^b^**
All-cause HCRU						
Any inpatient admission, n (%)	722 (18.29)	692 (28.31)	<.0001	296 (3.87)	396 (26.67)	.0273
Any ED visit, n (%)	905 (22.92)	834 (34.12)	<.0001	322 (33.58)	512 (34.48)	.6623
PCP visit, n (%)	3513 (88.98)	2318 (94.84)	<.0001	893 (93.12)	1425 (95.96)	.0026
Specialist visit, n (%)						
Oncologist	2889 (73.18)	1838 (75.20)	.0737	772 (8.50)	1066 (71.78)	<.0001
Surgeon	2200 (55.72)	1466 (59.98)	.0009	602 (62.77)	864 (58.18)	.0250
Psychiatrist	8 (0.20)	130 (5.32)	<.0001	25 (2.61)	105 (7.07)	<.0001
Other specialists	3747 (94.91)	2377 (97.26)	<.0001	932 (97.18)	1445 (97.31)	.8993
Overall	3847 (97.44)	2423 (99.14)	<.0001	952 (99.27)	1471 (99.06)	.6585
LOS, mean (SD)	1.06 (3.99)	2.23 (6.28)	<.0001	2.26 (5.83)	2.21 (6.56)	.0597
Any mental health services	29 (0.73)	407 (16.65)	<.0001	149 (15.54)	258 (17.37)	.2433

Psychiatric disorders include anxiety, depression, and stress reaction/adjustment disorders.

Abbreviations: ED, emergency department; HCRU, healthcare resource utilization; LOS, length of stay; PCP, primary care provider/physician.

^a^No psychiatric disorders vs psychiatric disorders.

^b^Incident vs prevalent.

When comparing patients with new onset psychiatric disorders (ie, incident) vs those with pre-existing psychiatric disorders (ie, prevalent), patients with incident psychiatric disorders had higher rates of inpatient admissions (30.87% vs 26.67%, *P* = .0273), whereas patients with prevalent psychiatric disorders had higher rates of PCP visits (95.96% vs 93.12%, *P* = .0026). Rates of ED visits were similar between these 2 patient groups, as was average LOS for inpatient admissions. Regarding specialty care, overall rates of specialist visits were similar, but patients with prevalent psychiatric disorders had higher rates of psychiatry visits (7.07% vs 2.61%, *P* < .0001). Rates of any mental health services were similar between these 2 patient groups (**Table 1**).

During the first year following breast cancer diagnosis, patients with anxiety, depression, or stress reaction/adjustment reaction incurred significantly higher average costs for ED visits ($1146 [2806] vs $598 [1936] per patient per year [PPPY]; *P* < .0001), inpatient admissions ($8394 [23 123] vs $4496 [15 671] PPPY; *P* < .0001), PCP visits ($1082 [4398] vs $794 [6561] PPPY; *P* < .0001), and specialist visits ($8738 [23 938] vs $6846 [21 963] PPPY; *P* < .0001) compared with patients without these psychiatric disorders. On average, total medical costs ($65 594 [90 999] vs $52 212 [78 397] PPPY; *P* < .0001), total pharmacy costs ($6628 [25 091] vs $5199 [19 797] PPPY; *P* < .0001), and total healthcare costs ($72 223 [94 646] vs $57 411 [81 088] PPPY; *P* < .0001) were significantly higher for patients with anxiety, depression, or stress reaction/adjustment disorder than patients without these psychiatric disorders (**Table 2**).

**Table 2.** Healthcare Costs Among Patients with Breast Cancer in the First Year Following Diagnosis

	**No Psychiatric Disorders** **(n = 948)**	**Psychiatric Disorders (n= 2444)**	***P* Value** ^a^	**Psychiatric Disorders**		
				**Incident (n = 959)**	**Prevalent (n = 1485)**	***P* Value^b^**
All-cause HCRU cost ($), mean (SD)						
Inpatient admissions	4496 (15 671)	8394 (23 123)	<.0001	9347 (24 240)	7778 (22 358)	.0232
ED visit	598 (1 936)	1146 (2 806)	<.0001	1282 (3 310)	1058 (2 423)	.8604
PCP visit	794 (6 561)	1082 (4 398)	<.0001	946 (2 912)	1170 (5 133)	.0010
Specialist visit	6846 (21 963)	8738 (23 938)	<.0001	10 914 (28 831)	7333 (20 042)	<.0001
Total medical						
Breast cancer chemotherapy	5830 (30 598)	8053 (38 297)	<.0001	11 838 (51 699)	5608 (25 997)	<.0001
Breast cancer radiotherapy	3491 (11 193)	3655 (12 861)	.0225	4201 (14 430)	3302 (11 729)	.4008
Breast cancer immunotherapy	2008 (17 213)	2549 (19 163)	.1762	3674 (24 034)	1822 (15 174)	.0388
Breast cancer surgery	4792 (6 381)	5547 (7 535)	.0005	6403 (8481)	4995 (6 800)	<.0001
Other surgery	4054 (7 027)	5263 (8 942)	<.0001	6285 (8832)	4602 (8 254)	<.0001
Pain/fatigue/nausea/vomiting-related costs	85 (675)	246 (1 832)	<.0001	262 (1692)	235 (1901)	.9257
Any mental health services	33 (259)	708 (2 616)	<.0001	459 (1554)	868 (3105)	<.0001
Other medical costs	31 916 (52 003)	39 517 (57 647)	<.0001	45 970 (61 720)	35 439 (54 478)	<.0001
Overall	52 212 (78 397)	65 594 (90 999)	<.0001	79 096 (105 686)	56 875 (78 915)	<.0001
Total pharmacy	5199 (19 797)	6628 (25 091)	<.0001	6438 (22 513)	6751 (26 631)	<.0001
Total healthcare costs	57 411 (81 088)	72 223 (94 646)	<.0001	85 535 (108 568)	63 627 (83 361)	<.0001

Psychiatric disorders include anxiety, depression, and stress reaction/adjustment disorders.

Abbreviations: ED, emergency department; HCRU, healthcare resource utilization; PCP, primary care provider/physician.

^a^No psychiatric disorders vs psychiatric disorders.

^b^Incident vs prevalent.

When comparing patients with incident and prevalent psychiatric disorders, patients with incident psychiatric disorders incurred higher average costs for inpatient admissions ($9347 [24 240] vs $7778 [22 358] PPPY; *P* = .0232) and specialist visits ($10 914 [28 831] vs $7333 [20 042] PPPY; *P* < .0001), whereas patients with prevalent psychiatric disorders incurred higher average costs for PCP visits ($1170 [5133] vs $946 [2912] PPPY; P = .0010). Average costs for ED visits were similar. On average, total medical costs ($79 096 [105 686] vs $56 875 [78 915] PPPY; *P* < .0001) and total healthcare costs ($85 535 [108 568] vs $63 627 [83 361] PPPY; *P* < .0001) were significantly higher for patients with incident psychiatric disorders, whereas total pharmacy costs ($6751 [26 631] vs $6438 [22 513] PPPY; *P* < .0001) were significantly higher for patients with prevalent psychiatric disorders (**Table 2**).

Regarding mental health–related costs, patients with anxiety, depression, or stress reaction/adjustment disorder incurred higher average costs for any mental health services than patients without these psychiatric disorders ($708 [2616] vs $33 [259] PPPY; *P* < .0001). Among patients with anxiety, depression, or stress reaction/adjustment disorder, patients with prevalent psychiatric disorders incurred higher average costs for any mental health services than patients with incident psychiatric disorders ($868 [3105] vs $459 [1554] PPPY; *P* < .0001; **Table 2**).

All-cause HCRU and costs for incident and prevalent anxiety, depression, and stress reaction/adjustment disorder are provided in **Supplementary Table S2**.

### Objective 3: Association Between Healthcare Costs and Anxiety, Depression, and Stress Reaction/Adjustment Disorder

**Figure 3.**
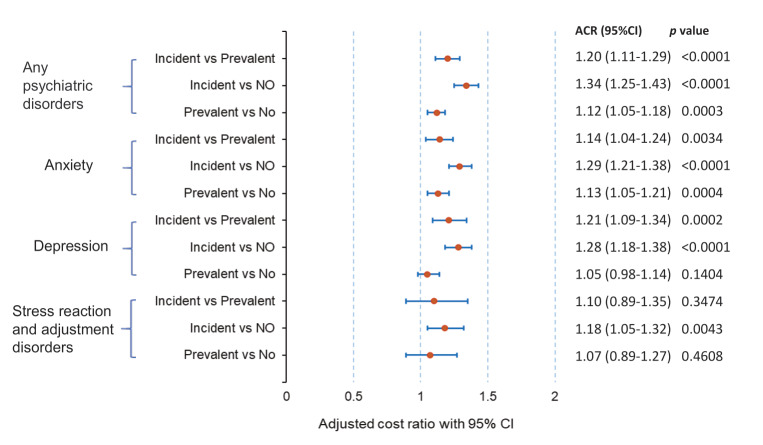
Multivariable Generalized Linear Regression: Adjusted Cost Ratios Multivariable adjusted cost ratios for total all-cause healthcare costs among newly diagnosed patients with breast cancer. Adjusted covariables included age, geographic region, rural/suburban/urban residence, household income, payer type, surgery year, number of comorbid conditions, number of cancer-related therapies, and previous year’s total all-cause healthcare cost. Any psychiatric disorders include anxiety, depression, and stress reaction/adjustment disorders.

**Figure 4.**
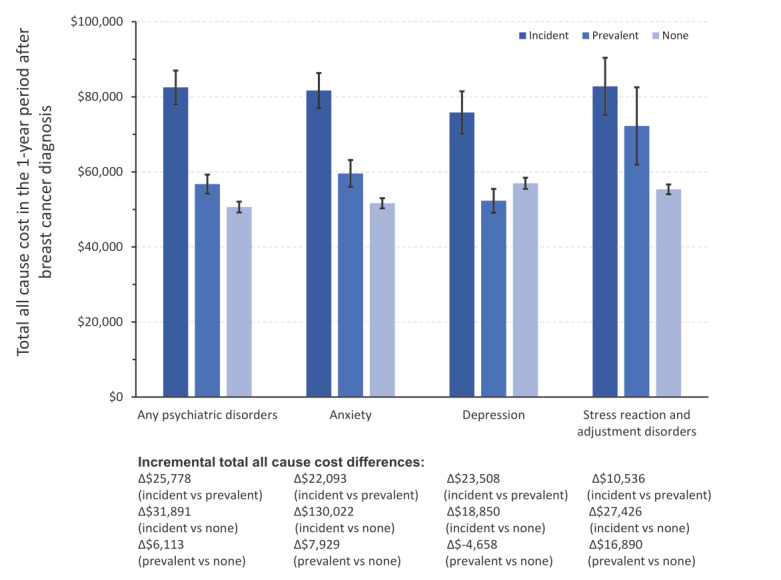
Multivariable Generalized Linear Regression: Incremental Total All-Cause Cost Differences

Patients with breast cancer who had incident anxiety, depression, or stress reaction/adjustment disorder incurred higher all-cause healthcare costs in the first year following breast cancer diagnosis than those with prevalent anxiety, depression, or stress reaction/adjustment disorder, or those with none of these psychiatric disorders (adjusted cost ratio: 1.20 [1.11-1.29], *P* < .0001, 1.34 [1.25-1.43], *P* < .0001, respectively; **Figure 3**). The predicted incremental total all-cause cost difference in the 1-year period after breast cancer diagnosis between incident psychiatric disorders and prevalent psychiatric disorders was $25 778 PPPY, and the incremental total all-cause cost difference between incident psychiatric disorders and no psychiatric disorders was $31 891 PPPY (**Figure 4**). A similar trend was also observed when considering anxiety, depression, and stress reaction/adjustment disorder individually (**Figures 3 and 4**).

## DISCUSSION

Our study included a large and representative sample, drawn from one of the largest national commercially insured and Medicare Advantage populations in the United States, representing over 20 million enrollees and providing a 1-year baseline and 1-year follow-up period. To our knowledge, this study is the first to assess the incidence and prevalence of anxiety, depression, and stress reaction/adjustment disorder among patients newly diagnosed with breast cancer and their impact on HCRU and costs. According to our study, a significant proportion of patients experienced anxiety, depression, or stress reaction/adjustment disorder during the first year following breast cancer diagnosis. Although rates of anxiety, depression, and stress among patients with breast cancer vary widely throughout the literature, our findings suggest that the rate of anxiety observed in the present study was comparable to estimates for early-stage breast cancer,^6^ whereas depression and stress reaction/adjustment disorder were observed in a smaller proportion of patients in the present study.

A significantly higher proportion of patients with anxiety, depression, or stress reaction/adjustment disorder had inpatient admissions, ED visits, PCP visits, psychiatry visits, mental health services visits, or other specialist visits, and significantly longer LOS compared with patients without these psychiatric disorders. Similarly, the costs of ED visits, inpatient admissions, PCP visits, specialist visits, and total medical and pharmacy expenses were higher for patients with anxiety, depression, or stress reaction/adjustment disorder compared with patients without these psychiatric disorders. Overall, anxiety, depression, and stress reaction/adjustment disorder were associated with increased costs among patients with breast cancer, as evidenced by significantly higher all-cause healthcare costs with the exception of prevalent depression.

Patients with breast cancer who experienced incident anxiety, depression, or stress reaction/adjustment disorder had higher all-cause healthcare costs than those with prevalent diagnoses of these psychiatric disorders or those without these disorders in the first year following breast cancer diagnosis. The higher healthcare costs incurred by patients with incident psychiatric disorders compared with prevalent psychiatric disorders suggest that new-onset anxiety or depression may contribute to higher costs incurred by the payer. Early psychosocial intervention has not only been found to be effective in improving clinical outcomes, but also leads to cost savings in the long term.^29^

The comparison of patients with incident and prevalent anxiety, depression, and stress reaction/adjustment disorder yielded interesting findings regarding potential treatment patterns. Specifically, patients with prevalent psychiatric disorders had higher rates of PCP visits and psychiatry visits compared with patients with incident psychiatric disorders. These results suggest that patients with pre-existing psychological distress may have already established care with a PCP or psychiatrist for medication management. Although rates of any mental health services were similar between these 2 patient groups, patients with prevalent psychiatric disorders incurred higher average costs for any mental health services. This may suggest that both patients with pre-existing and new-onset psychological distress are equally amenable to engaging in mental health services following breast cancer diagnosis, but patients with prevalent psychiatric disorders may seek care more frequently or may have more complex visits, resulting in higher costs. Additionally, total pharmacy costs were significantly higher for patients with prevalent psychiatric disorders, suggesting again that these patients may have already established care for medication management, resulting in higher total pharmacy costs.

Overall, patients who experienced anxiety, depression, or stress reaction/adjustment disorder following breast cancer diagnosis may represent a vulnerable population with unmet needs. Left untreated, psychological distress in patients with cancer could increase the morbidity of the disease and negatively affect patients’ clinical outcomes and survival.^10,11^ Furthermore, distress and psychiatric disorders have an impact on treatment engagement and effectiveness, which in turn may increase HCRU and economic burden.^10,11^

To diminish the burden of psychological distress, improve clinical outcomes, and reduce healthcare costs, viable treatment options for psycho-oncological distress are needed. In addition to addressing the unmet needs of patients with breast cancer who experience new-onset psychiatric disorders, effective treatments are also needed for patients whose pre-existing psychiatric disorders may be exacerbated by breast cancer diagnosis and treatment. Moreover, multiple comorbidities are common among patients with breast cancer and are associated with increased risk of anxiety and depression in the first year following breast cancer diagnosis.^19^ Due to limited availability of specialized psychological care for patients with cancer,^17,18^ there is a growing need for new treatment modalities, such as digital therapeutics. A recent review suggests that digital therapeutics in oncology possess clinical value, provide benefits perceived by both patients and healthcare providers, and may have a positive impact on HCRU, although further research is needed.^30^

In addition, future studies are warranted to support the early identification of newly diagnosed patients with breast cancer who are at elevated risk of clinically meaningful psychological distress within the first year following diagnosis. While there is no algorithm or predictive model currently available to identify patients with breast cancer who are at high risk of anxiety, depression, or stress reaction/adjustment disorder, the results of this study may inform the development of a tool to aid clinicians in the early identification of at-risk patients, providing an opportunity to improve clinical outcomes and reduce HCRU and costs. Other avenues for future research could include the exploration of different methodologies and their impact on results. For example, whereas the present study utilized the widely used Charlson Comorbidity Index, future research may consider using a cancer-specific comorbidity index, such as the National Cancer Institute comorbidity index.^31,32^

It is important to note that all studies using healthcare administrative claims data have inherent limitations. To begin with, only Aetna plan members were included in the present study. Consequently, the results of this analysis and their implications may not be generalizable to the overall population, especially those with other types of health insurance and the uninsured population. Next, there may be certain information not available in claims data that could have influenced study outcomes, such as clinical and disease-specific parameters, which may result in residual confounding. For example, disease severity could explain the outcomes of the present study in that late-stage breast cancer could result in increased psychological distress, increased HCRU, and increased spending. Additionally, although high out-of-pocket costs constitute financial toxicity to patients,^33^ our study did not capture healthcare costs incurred by patients. Patients may not have accessed mental health care due to high costs or other more urgent medical conditions.

Another limitation of the present study is that anxiety, depression, and stress reaction/adjustment disorder were identified using ICD-10-CM codes only. Therefore, the incidence and prevalence of these disorders may have been underestimated. Additionally, the 1-year baseline period utilized to classify patients as having no previous history of psychiatric disorders (ie, incident) may have excluded patients who were diagnosed prior to that time. Therefore, among patients in this study, the incidence of these disorders may have been overestimated to the extent that the prevalence may have been underestimated. A significant proportion of patients with cancer report psychological distress (30%), but only a third of them (10%) seek medical attention for psychological distress.^34^ Limited accessibility and availability of mental health services for patients with cancer is one reason why the proportion of patients seeking these services may be low.^35^ In addition, patients with cancer may not be interested in reporting psychological distress to their healthcare providers,^36^ or receiving psycho-oncological services for psychological distress.^37^

## CONCLUSIONS

Anxiety, depression, and stress reaction/adjustment disorder are common among patients with newly diagnosed breast cancer and are significantly associated with increased HCRU and expenditures in the first year following breast cancer diagnosis. Patients with breast cancer who experienced incident anxiety, depression, or stress reaction/adjustment disorder had higher all-cause healthcare costs in the year following their cancer diagnosis than those with prevalent anxiety, depression, or stress reaction/adjustment disorder or none of these psychiatric disorders. Early diagnosis and optimized management of anxiety, depression, and stress reaction/adjustment disorder in this vulnerable population could improve clinical outcomes and reduce HCRU and healthcare costs.

### Disclosures

D.D. and H.C. report employment by CVS Health Corp., Woonsocket, Rhode Island, which conducted research funded by Blue Note Therapeutics, Inc. S.Z. and M.M. report employment by and stock in Blue Note Therapeutics, Inc.

## Supplementary Material

Online Supplementary Material
