# Supplementary material for: Anxiety, Depression, and Stress Reaction/Adjustment Disorders and Their Associations with Healthcare Resource Utilization and Costs Among Newly Diagnosed Patients With Breast Cancer
Source: J Health Econ Outcomes Res. 2023 Mar 28;10(1):68–76. doi: 10.36469/001c.70238 (PMC10062496; doi:10.36469/001c.70238)

Journal of Health Economics and Outcomes Research

Online Supplementary Material

Anxiety, Depression, and Stress Reaction/Adjustment Disorders and Their Associations With Healthcare Resource Utilization and Costs in Patients With Newly Diagnosed Breast Cancer. *JHEOR*. **2023;10(1):68-76.** doi:10.36469/jheor.2023.70238

Table S1: Baseline Demographic and Clinical Characteristics Among Newly Diagnosed Patients With Breast Cancer by Psychiatric Disorders

Table S2: All-Cause Healthcare Resource Utilization and Costs Among Newly Diagnosed Patients With Breast Cancer

Figure S1: Patient Attrition

This supplementary material has been provided by the authors to give readers additional information about their work.

This is an open-access article distributed under the terms of the Creative Commons Attribution 4.0 International License (CCBY-4.0). View this license's legal deed at http://creativecommons.org/licenses/by/4.0 and legal code at http://creativecommons.org/licenses/by/4.0/legalcode for more information.

Table S1. Baseline Demographic and Clinical Characteristics Among Newly Diagnosed Patients With Breast Cancer by Psychiatric Disorders

Characteristics	Overall (N = 6392 [100%])	No Psychiatric Disorders (n =3948 [61.8%])	Psychiatric Disorders (n = 2444 [38.2])	*P* Value[a]	Psychiatric Disorders		
					Incident Psychiatric Disorders (n =959 [15%])	Prevalent Psychiatric Disorders (n = 1485 [23.2%])	*P* Value[b]
Age (y), mean (SD)	67.43 (12.23)	68.01 (11.98)	66.48 (12.56)	<.0001	64.55 (13.50)	67.73 (11.76)	<.0001
Geographic region, n (%)		.0106		.0124			
Midwest	1357 (21.23)	785 (19.88)	572 (23.40)		216 (22.52)	356 (23.97)	
Northeast	1967 (30.77)	1234 (31.26)	733 (29.99)		294 (3.66)	439 (29.56)	
South	2519 (39.41)	1585 (40.15)	934 (38.22)		355 (37.02)	579 (38.99)	
West	549 (8.59)	344 (8.71)	205 (8.39)		94 (9.80)	111 (7.47)	
Urban-rural, n (%)		<.0001		<.0001			
Urban	1811 (28.33)	1190 (30.14)	621 (25.41)		289 (3.14)	332 (22.36)	
Suburban	1951 (30.52)	1210 (30.65)	741 (30.32)		281 (29.30)	460 (3.98)	
Rural	2630 (41.15)	1548 (39.21)	1082 (44.27)		389 (4.56)	693 (46.67)	
Median household income ($), median (IQR)	60 102 (47 499-78 772)	61 041(47 560-79 883)	59 244 (47 419-77 222)	.1951	59 705 (47 332-79 078)	58 906 (47 427-75 758)	.3398
Payers, n (%)		.0485		<.0001			
Commercial insurance	1989 (31.12)	1264 (32.02)	725 (29.66)		349 (36.39)	376 (25.32)	
Medicare Advantage	4403 (68.88)	2684 (67.98)	1719 (70.34)		610 (63.61)	1109 (74.68)	
No. of comorbid conditions, mean (SD)	5.45 (3.55)	4.79 (3.25)	6.49 (3.75)		4.58 (3.22)	7.73 (3.55)	<.0001
CCI, mean (SD)	1.48 (1.91)	1.36 (1.83)	1.66 (2.00)	<.0001	1.34 (1.78)	1.87 (2.11)	<.0001
Age-adjusted CCI, mean (SD)	4.64 (2.56)	4.57 (2.47)	4.74 (2.69)	.0232	4.22 (2.59)	5.08 (2.69)	<.0001
All-cause HCCs ($), mean (SD)	11 419 (24 441)	9858 (20 665)	13 940 (29 368)	<.0001	9558 (16 611)	16 769 (34 948)	<.0001

Psychiatric disorders include anxiety, depression, and stress reaction/adjustment disorders.
Abbreviations: CCI, Charlson Comorbidity Index; HCCs, healthcare costs; IQR, interquartile range; SD, standard deviation.
[a]No psychiatric disorders vs psychiatric disorders.
[b]Incident vs prevalent.

Table S2. All-Cause Healthcare Resource Utilization and Costs Among Newly Diagnosed Patients With Breast Cancer

	**Anxiety**			**Depression**			**Stress Reaction and Adjustment Disorders**		
	**Incident (n = 864)**	**Prevalent (n = 909)**	***P* Value[a]**	**Incident (n = 615)**	**Prevalent (n = 787)**	***P* Value[a]**	**Incident (n = 272)**	**Prevalent (n = 110)**	***P* Value[a]**
All-cause HCRU, n (%)									
Any inpatient admission	259 (29.98)	265 (29.15)	.0715	217 (35.28)	210 (26.68)	.0006	88 (32.35)	29 (26.36)	.2717
Any ED visit	302 (34.95)	336 (36.96)	.4001	224 (36.42)	300 (38.12)	.5407	98 (36.03)	32 (29.09)	.2332
PCP visit	816 (94.44)	870 (95.71)	.2279	575 (93.50)	760 (96.57)	.0081	255 (93.75)	104 (94.55)	.9534
Specialist visit	856 (99.07)	903 (99.34)	.5981	609 (99.02)	778 (98.86)	.8008	268 (98.53)	110 (10.00)	.5822
LOS, mean (SD)	2.04 (5.22)	2.52 (7.34)	.8620	2.67 (6.26)	2.51 (7.72)	.6735	2.80 (6.73)	1.40 (4.04)	.1232
All-cause HCCs ($), mean (SD)									
Inpatient admissions	8853 (23 403)	9023 (25 434)	.6878	9 448 (21 857)	8 143 (24 774)	.0016	12 814 (33 642)	6837 (18 631)	<.0001
ED visit	1300 (3041)	1 46 (2607)	.6917	1263 (3153)	1178 (507)	.5276	1463 (3869)	989 (2342)	<.0001
PCP visit	916 (1980)	1334 (262)	.0112	1094 (5 465)	1017 (2466)	.1043	1 11 (2302)	2375 (12 558)	<.0001
Specialist visit	10 960 (28 478)	7817 (20 513)	.0002	10 026 (26 047)	6092 (16 807)	.0018	11 125 (28 289)	10 896 (29 004)	.7380
Total medical	78 316 (98 452)	61 315 (86 934)	<.0001	75 187 (105 701)	53 818 (75 467)	<.0001	85 281 (113 959)	77 350 (97 644)	.0634
Total pharmacy	6183 (21 223)	7515 (31 296)	<.0001	7575 (23 971)	7126 (31 773)	.0003	5583 (21 745)	5 572 (20 316)	.4150
Total healthcare costs	84 500 (101 632)	68 830 (92 341)	<.0001	82 762 (108 325)	60 945 (81 709)	<.0001	90 865 (116 924)	82 922 (99 365)	.0264

Abbreviations: ED, emergency department; HCCs, healthcare costs; HCRU, healthcare resource utilization; LOS, length of stay; PCP, primary care provider/physician, SD, standard deviation.
[a]Incident vs prevalent.

Figure S1. Patient Attrition

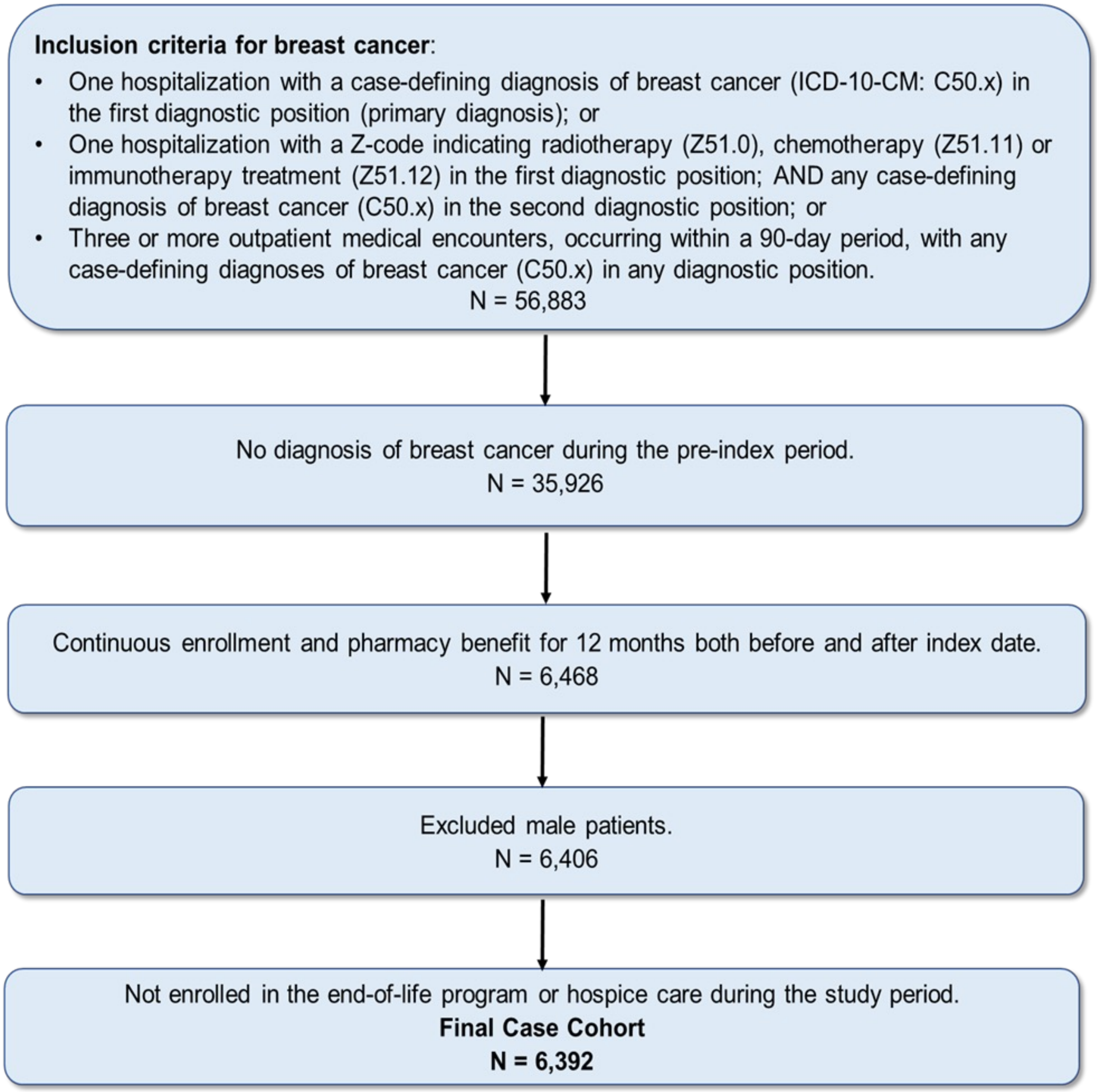

Supplement: Online Supplementary Material [file jheor_2023_10_1_70238_155064.pdf]
